# Effects of Mn content on austenite stability and mechanical properties of low Ni alumina-forming austenitic heat-resistant steel: a first-principles study

**DOI:** 10.1038/s41598-023-32968-9

**Published:** 2023-04-08

**Authors:** Yanjun Zhao, Yunfei Cao, Weiying Wen, Zepeng Lu, Jingrui Zhang, Yafei Liu, Peilin Chen

**Affiliations:** 1 College of Resources, Environment and Materials, Guangxi University, Nanning, 530004 China; 2 Key Laboratory of High Performance Structural Materials and Thermo-Surface Processing (Guangxi University), Education Department of Guangxi Zhuang Autonomous Region, Nanning, 530004 China

**Keywords:** Materials science, Mathematics and computing

## Abstract

Low Ni alumina-forming austenitic (AFA) heat-resistant steel is an advanced high-temperature stainless steel with reduced cost, good machinability, high-temperature creep strength, and high-temperature corrosion resistance. Using the First-principles approach, this study examined the effect of Mn content on austenite stability and mechanical properties at the atomic level. Adding Mn to low Ni-AFA steel increases the unit cell volume with an accompanying increase in the absolute value of formation energy; the austenite formed more easily. The austenitic matrix binding energy decreases and remains negative, indicating austenite stability. As the Mn content increases from 3.2 to 12.8 wt%, the system's bulk modulus (B) rises significantly, and the shear modulus (G) falls. In addition, the system's strength and hardness decrease, and the Poisson ratio of the austenite matrix increases with improved elasticity; the system has excellent plasticity with an increase in the B/G. For the Fe_22_–Cr_5_–Ni_3_–Al_2_ system, with the increase of Mn content, the electron density distribution between the atoms is relatively uniform, and the electrons around the Mn atoms are slightly sparse, which will slightly reduce the structural stability of the matrix. The experiment demonstrated the matrix maintains the austenitic structure when adding 3.2–12.8 wt% Mn elements to low Ni-AFA steel. At an Mn content of 8 wt%, the overall mechanical properties of the high-Mn AFA steel are optimal, with a tensile strength of 581.64 MPa, a hardness of 186.17 HV, and an elongation of 39%.

## Introduction

Alumina-forming austenitic (AFA) steel is an advanced high-temperature-resistant stainless steel. The steel forms Al_2_O_3_ and Cr_2_O_3_ double oxide films at high temperatures, adding 1.5–3.5 wt% Al^1^. AFA steel has excellent high-temperature creep strength and corrosion resistance at 500–950 °C, 50–200 °C higher when compared with standard heat-resistant steels having only the Cr_2_O_3_ layer. AFA steel has potential applications in power generation, petrochemical, and energy fields, especially as the core component of steam engines in ultra-supercritical units^2^. Under long-term high-temperature and high-pressure service conditions, the steel must have a stable austenitic matrix to sustain high-temperature oxidation resistance and other crucial mechanical properties.

A high Ni content is required to counteract the effect of ferrite-forming elements such as Al and Cr to obtain stable and single austenite. However, the cost and scarcity of Ni have limited the application of austenitic steels^3^. Replacing Ni with inexpensive elements, such as C, N, Mn, and Cu, can reduce nickel consumption while maintaining high-temperature oxidation resistance and mechanical properties. Mn is the preferred substitution element as it is a strong austenitic stabilizer and much cheaper than Ni, significantly reducing the overall production cost^4–6^. However, Mn tends to form MnS compounds, leading to a deterioration of the corrosion properties^7–9^. In addition, a high Mn content lowers the steel weldability^10^, so an appropriate Mn content is critical in partial substitution for Ni^11^.

First-principles methods based on density functional theory can be applied to predict steel's mechanical, corrosion, and interfacial properties at the atomic level^12^. Yang et al.^13^ studied the electronic structure and elastic properties of metal element-doped α-Fe (N) high-nitrogen steel through first-principles methods. They verified that Mn and Ni slightly weakened the stability of α-Fe (N), enhancing the overall elastic performance. Wang et al.^14^ carried out a first-principles study on the stacking fault energy of Fe–Mn alloy and found that Mn atoms have an obvious short-range effect on the stacking fault energy in the matrix. Adding Co and W to Sanicro 25 austenitic heat-resistant steel can improve structural and thermodynamic stability^15^. Huang et al.^16^ investigated the effects of alloying elements on the structural stability and segregation behavior of the γ-Fe(111)/Cr_2_N(0001)interface using first principles. They concluded that Mn reduced the local electrochemical corrosion behavior of the γ-Fe/Cr_2_N interface by reducing the voltage potential difference (VPD) between them. Dong et al.^17^ studied the effect of Al on the composition optimization and mechanical properties of an AFA heat-resistant steel. When Al was present as a solid solution in the Fe–Cr–Ni alloy system, the austenitic matrix was stable at high temperatures; the solid solution of Al and Al + Si improved the system's plasticity. It is essential and rare to study the influence of altering the Mn content in Mn-substituted-for Ni AFA steel on the structural stability and mechanical properties by the first-principles method at an atomic scale.

In this study, an Mn-substituted-for Ni AFA steel (Ni content reduced from 20 wt% of traditional AFA steel to 10 wt%) with high Mn content (up to 12.8 wt%) is used as a means of reducing costs. The lattice parameters, formation energy, binding energy, elastic constant, ideal stress–strain curve, and state density are calculated using the first-principles method. We explored the strength of Mn-substituted-for Ni AFA steel and assessed the influence of Mn content (3.2–12.8 wt%) on the structural stability and mechanical properties at the atomic level. The optimal mechanical properties are demonstrated experimentally, which can inform the production of high-performance heat-resistant steel for the core component of steam engines in ultra-supercritical units.

## Computational method and material structure

### Calculation methods

**Figure 1:**
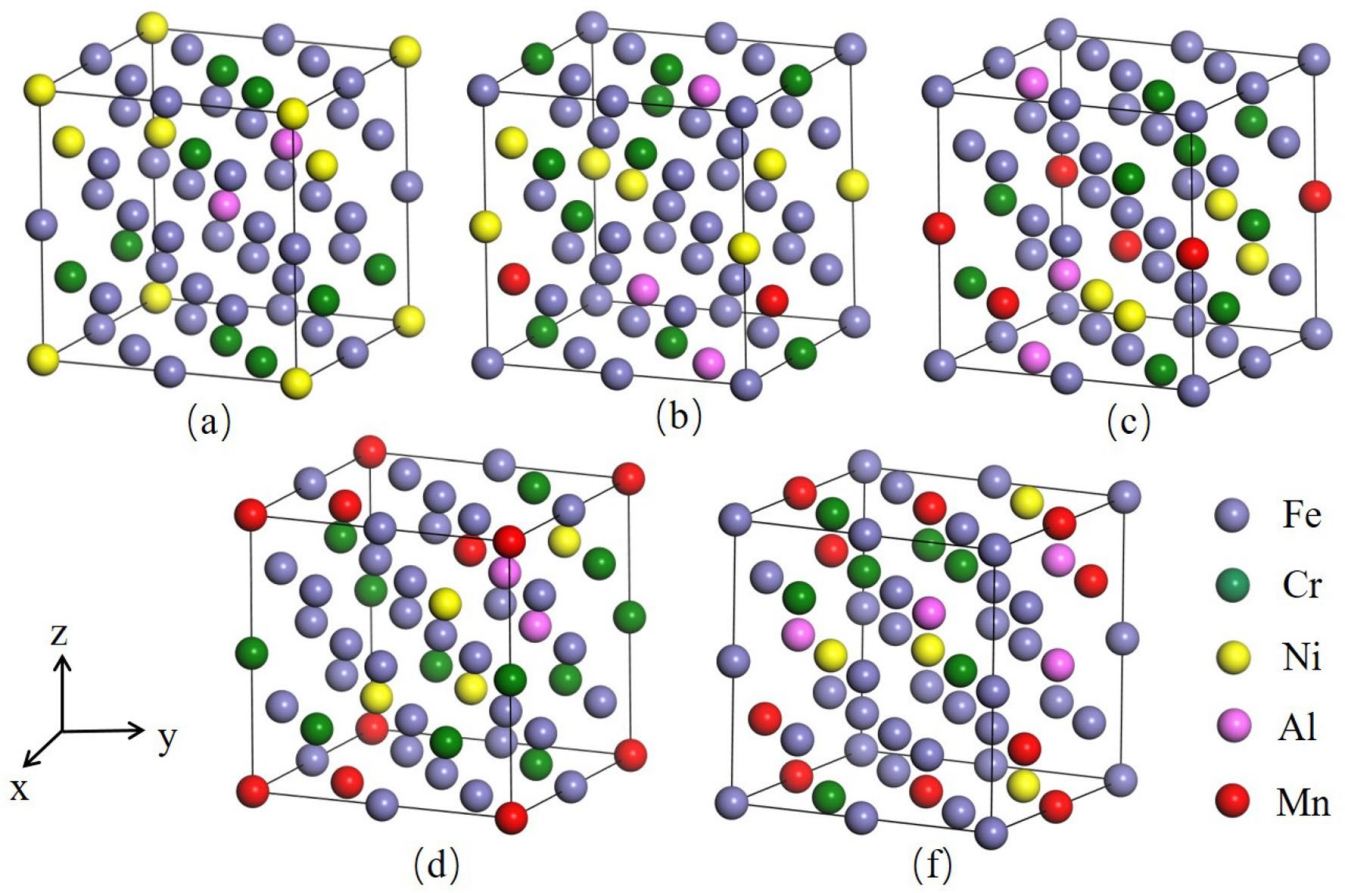
Schematic diagram of the calculation model: (**a**) Fe_22_–Cr_5_–Ni_3_–Al_2_ (**b**) Fe_21_–Cr_5_–Ni_3_–Al_2_–Mn (**c**) Fe_20_–Cr_5_–Ni_3_–Al_2_–Mn_2_ (**d**) Fe_19_–Cr_5_–Ni_3_–Al_2_–Mn_3_ (**e**) Fe_18_–Cr_5_–Ni_3_–Al_2_–Mn_4_.

The designed high-Mn AFA steel is Fe–14Cr–10Ni–3Al– (3.2–12.8) Mn (wt%) with Cr, Ni, Al, and Mn as the main alloying elements, and other alloying elements such as Nb, Cu, Ti, and Si also be added. As austenitic steel, γ-Fe has an Fcc structure, belonging to a cubic crystal system with the space group Fm-3 m. Atomic sizes of Cr, Ni, Al, and Mn are close to Fe atoms and are mainly present in the matrix as a solid solution. The modeling idea is to add different contents of Mn into the basis alloy system Fe–14Cr–10Ni–3Al (wt%). Considering the number of atoms and the actual computational efficiency, 2 × 2 × 2 supercells containing 32 atoms were used to represent the matrix structure of the high-Mn AFA steel. We used the mcsqs (Monte Carlo special quasirandom structure) algorithm based on the special quasi-random structure model in the Disordered Alloy Theory Research Toolkit (ATAT) to construct a reasonable crystal structure model of Fe_22_–Cr_5_–Ni_3_–Al_2_. We found the closest random model by matching the cluster association function and then simulated the disordered structure of Fe_22_–Cr_5_–Ni_3_–Al_2_ (The calculated model is shown in Fig. 1). The Fe_22_–Cr_5_–Ni_3_–Al_2_ model was constructed based on Fe–14Cr–10Ni–3Al system (Fig. 1a). Considering the computational efficiency, the composition percentage of the model is close to the composition of Fe–14Cr–10Ni–3Al (wt%). Then Mn atoms were added into the Fe_22_–Cr_5_–Ni_3_–Al_2_ model (Fig. 1a) to obtain Fig. 1b–e models, where the proportions of Mn atoms (3.125, 6.25, 9.375, and 12.5 at.%) converted to weight percentages were 3.2, 6.4, 9.6, and 12.8 wt%, respectively.

**Figure 2:**
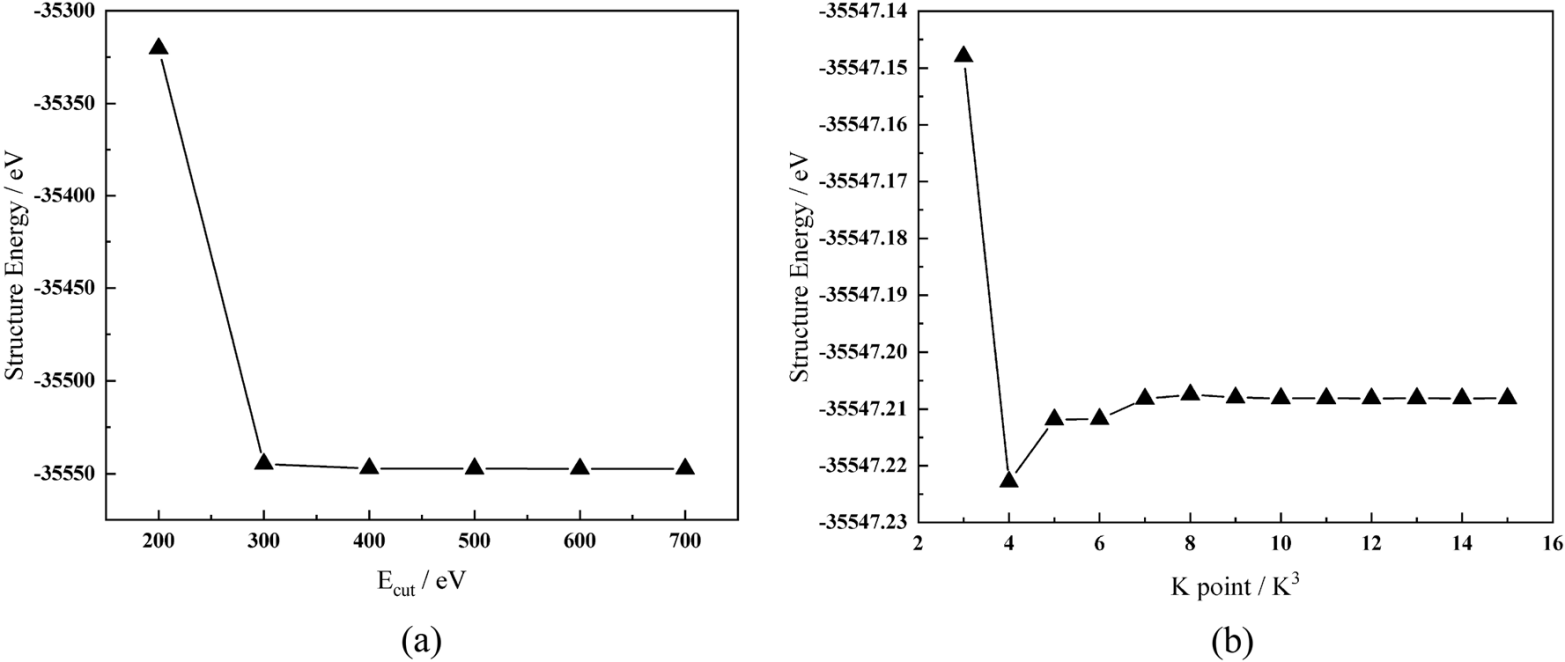
Convergence tests of the parameters: (**a**) Energy cut-off (E_cut_) (**b**) K Point.

The first-principles calculations were based on the CASTEP (Cambridge serial total energy package) module in MS (Material Studio) software for the models given in Fig. 1. The calculation parameters were as follows: the approximation of commutative associative energy selected the PBE (Pardew–Burke–Engenho) function under the generalized gradient approximation; the Boyden–Fletcher–Goldfarb–Shannon (BFGS) method was used for self-consistent field operations; the valence electron potential field was constructed by ultrasoft pseudopotential^18^. Convergence tests were performed for the K-point and cut-off energy (E_cut_), as shown in Fig. 2. When the E_cut_ and the K-point are 400.0 eV and 8 × 8 × 8, respectively, the system's energy converges. That means when the E_cut_ was 400.0 eV, the K point of the unit cell in the Brillouin region was taken as 8 × 8 × 8. The convergence accuracy of the self-consistent field was set to 1 × 10^−5^ eV/atom. After structural optimization, the force on each atom was less than 0.3 eV/nm, the tolerance offset less than 1 × 10^–4^ nm, and the stress deviation 0.05 GPa. In this study, austenitic heat-resistant steel contains Fe, Cr, Ni, Mn, and other atoms, and the steel exhibits weak magnetism. Considering the spin polarization approximation, the antiferromagnetic of double layers was added to the model's calculation after the convergence tests.

### Materials and characterization

**Figure 3:**
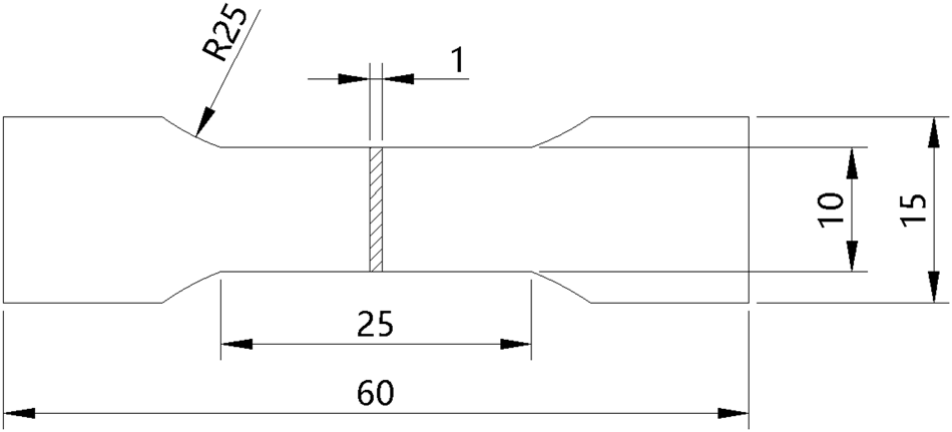
Schematic diagram of tensile specimen (mm).

Table 1 shows the chemical composition of the tested steel. The steel was received as a hot-rolled plate with a 2.1 mm thickness. The hot-rolled plate was solution treatment at 1150 °C for 2.5 h, air-cooled to room temperature, and then cold-rolled to 1.0 mm thickness. The tensile specimen was designed according to GB/T 228.1-2010, as shown in Fig. 3. A one-way tensile experiment was performed at room temperature using the INSTRON-8801 testing machine with a tensile rate of 2 mm/min. A microhardness test was performed using the HVS-1000 digital microhardness tester with a loading force of 4.903 N and a loading time of 10 s. The tensile and microhardness tests took the average value of five and nine measurement results, respectively.

**Table 1:** The compositions of high-Mn AFA steel (wt%).

Steels	C	Cr	Ni	Al	Mn	Cu	Nb	Si	S	P	Ti	Fe
6Mn	0.11	13.95	10.08	2.58	6.03 (5.91 at.%)	3.01	0.84	0.55	0.0034	< 0.001	0.051	Bal
8Mn	0.12	13.93	10.08	2.52	8.00 (7.85 at.%)	3.02	0.83	0.49	0.0035	< 0.001	0.051	Bal
10Mn	0.12	13.96	10.25	2.46	10.26 (10.08 at.%)	3.07	0.85	0.36	0.0024	< 0.001	0.048	Bal
12Mn	0.12	14.00	10.13	2.51	12.02 (11.82 at.%)	3.02	0.83	0.21	0.0025	< 0.001	0.049	Bal

## Results and discussion

### Effect of Mn content on austenitic stability of low Ni AFA steel

#### Effect of formation energy and binding energy on austenite stability of low Ni AFA steel with different Mn contents

The structural parameters of Fe_22_–Cr_5_–Ni_3_–Al_2_, Fe_21_–Cr_5_–Ni_3_–Al_2_–Mn, Fe_20_–Cr_5_–Ni_3_–Al_2_–Mn_2_, Fe_19_–Cr_5_–Ni_3_–Al_2_–Mn_3_, and Fe_18_–Cr_5_–Ni_3_–Al_2_–Mn_4_ were calculated and are given in Table 2. After geometric optimization, all five belong to orthogonal crystal systems where a ≠ b ≠ c. An increase in Mn atoms solidified in the matrix accompanied by a volume expansion.

**Table 2:** Optimized parameters of the five structural systems.

Structure	α = β = γ/°	a/Å	b/Å	c/Å	V/Å^3^
Fe_22_–Cr_5_–Ni_3_–Al_2_	90	7.050833	7.06462	7.025765	349.963583
Fe_21_–Cr_5_–Ni_3_–Al_2_–Mn	90	7.111288	7.066416	6.987573	351.134762
Fe_20_–Cr_5_–Ni_3_–Al_2_–Mn_2_	90	7.012975	7.078049	7.077811	351.3296613
Fe_19_–Cr_5_–Ni_3_–Al_2_–Mn_3_	90	7.0256	7.067415	7.114321	353.246177
Fe_18_–Cr_5_–Ni_3_–Al_2_–Mn_4_	90	7.043309	7.051263	7.121751	353.696238

The formation energy and binding energy can be used to describe the stability of austenite in the five systems for high-Mn AFA steel. The formation energy measures the difficulty of forming the five systems^19^. The binding energy indicates that the multi-atomic systems must overcome certain attractive forces when they are joined together, reflecting the system structure's tightness^20^. The formation and binding energy can be calculated using Eqs. (1) and (2), respectively^19,21^.

1 $$ E_{f} = \frac{1}{{\sum N_{i} }}\left[ {E_{total} - \sum \left( {N_{i} E_{atom} } \right)} \right] $$

2 $$ E_{b} = \frac{1}{{\sum N_{i} }}\left[ {E_{total} - \sum \left( {N_{i} E_{iso} } \right)} \right] $$

where E_total_ is the total energy after optimization, N_i_ is the number of atoms i (i = Fe, Cr, Ni, Al, or Mn) in the unit cell, E_atom_ is the single atomic energy of atom i in its elemental state (the elemental states of Fe, Cr, Ni, Al and Mn are Fcc-Fe, Bcc-Cr, Fcc-Ni, Fcc-Al and Bcc-Mn in the computational state, respectively), and E_iso_ is the energy of atoms i in the isolated state, placing atom i in the center of a simple cubic structure of 10 Å and calculating its relaxed energy.

**Figure 4:**
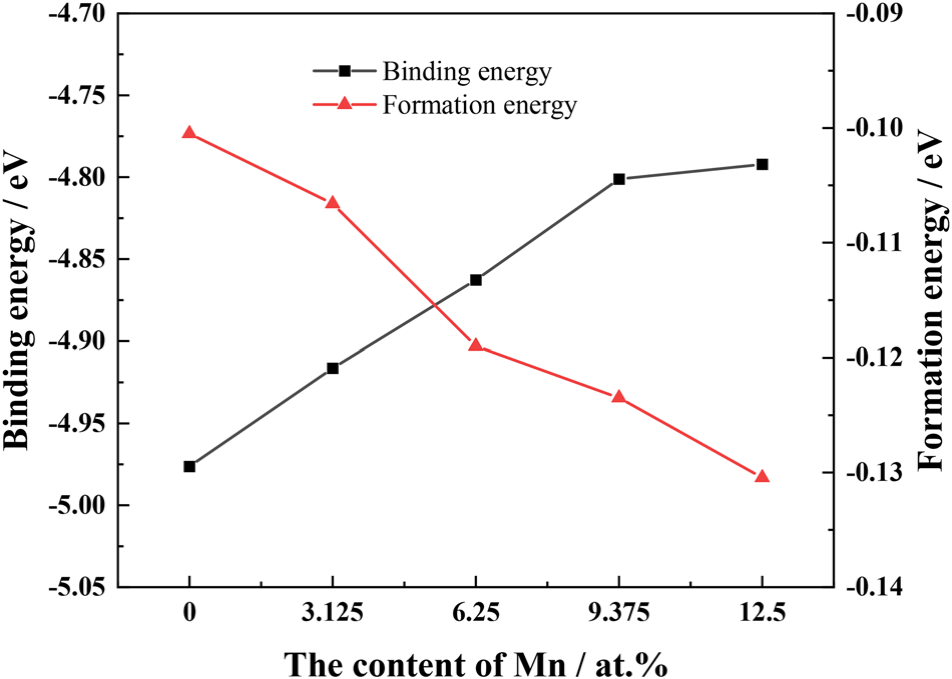
The effect of the Mn content on the formation energy and binding energy.

The formation and binding energy of Mn elements solidified in the Fe_22_–Cr_5_–Ni_3_–Al_2_ system are shown in Fig. 4. The formation energy for Fe_22_–Cr_5_–Ni_3_–Al_2_, Fe_21_–Cr_5_–Ni_3_–Al_2_–Mn, Fe_20_–Cr_5_–Ni_3_–Al_2_–Mn_2_, Fe_19_–Cr_5_–Ni_3_–Al_2_–Mn_3_, and Fe_18_–Cr_5_–Ni_3_–Al_2_–Mn_4_ are all negative value, indicating that all five structures can form stably and spontaneously. Fe_18_–Cr_5_–Ni_3_–Al_2_–Mn_4_ is the easiest to develop with increased Mn content dissolved in the Fe_22_–Cr_5_–Ni_3_–Al_2_ system. The absolute value of the formation energy of the system increases with the rising Mn content, and the entire austenitic structure is more easily formed. The binding energy of the five systems is between − 4.95 and − 4.75 eV, indicating that the five systems are very stable. An increase in Mn content in the solid solution with Fe_22_–Cr_5_–Ni_3_–Al_2_ was accompanied by a slight decrease in the absolute value of the binding energy, reflecting a limited effect on the stability of the structure, and the austenite can be stabilized. In summary, after adding Mn, the high-Mn AFA steel structure maintained stability, retaining the austenitic structure in the matrix.

#### Effect of electronic properties on the austenite stability of low Ni AFA steel with different Mn contents

**Figure 5:**
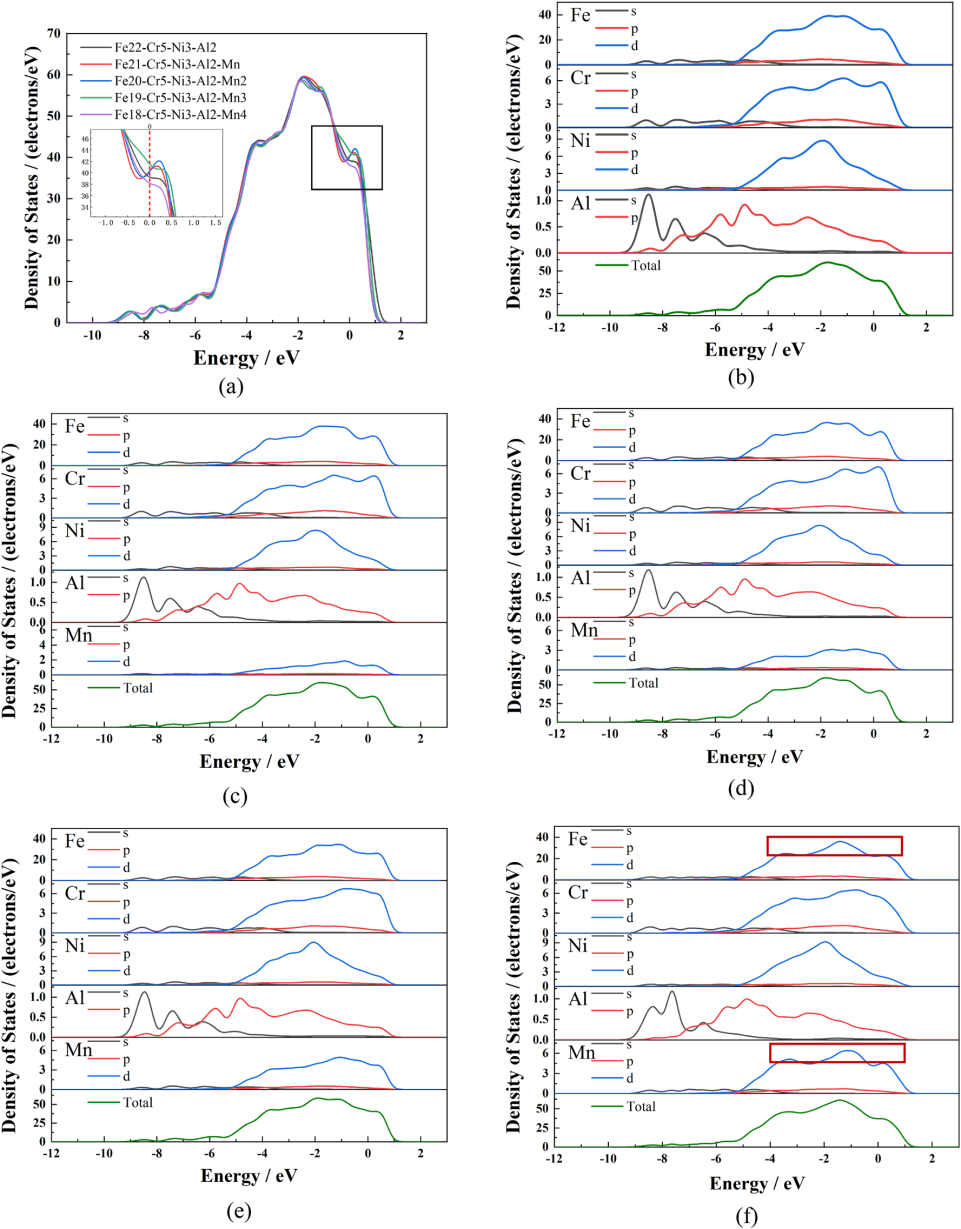
Total density of states and partial density of states of the five structural systems: (**a**) Total density of states (**b**) Fe_22_–Cr_5_–Ni_3_–Al_2_ (**c**) Fe_21_–Cr_5_–Ni_3_–Al_2_–Mn (**d**) Fe_20_–Cr_5_–Ni_3_–Al_2_–Mn_2_ (**e**) Fe_19_–Cr_5_–Ni_3_–Al_2_–Mn_3_ (**f**) Fe_18_–Cr_5_–Ni_3_–Al_2_–Mn_4_.

Figure 5a gives the total state density of five structural systems. The state density curves of the different systems are essentially similar, and most of the regions coincide, indicating that adding Mn did not change the energy level structure of the Fe_22_–Cr–Ni_53_–Al_2_ system. The electron distribution near the Fermi surface mainly determines the steel's properties. When different levels of Mn form a solid solution in the Fe_22_–Cr_5_–Ni_3_–Al_2_ system, the number of electrons at the Fermi energy level is non-zero. It exhibits metallic characteristics that increase first and then decrease. As the Mn content increased to 9.6 wt%, the electrochemical stability of the matrix increased, and when the Mn content further increased to 12.8 wt%, the electrochemical stability of the matrix decreased.

Taking the Fe_22_–Cr_5_–Ni_3_–Al_2_ system, the energy levels below the Fermi energy level are mainly due to the 3d orbital electrons of Fe, with a secondary contribution from the 2p orbital electrons of Cr, the 3d orbital electrons of Ni and the 1s and 2p orbital electrons of Al. Energy levels above the Fermi are primarily attributed to the 3d orbital electrons of Fe, the 3d orbital electrons of Ni, and 2p orbital electrons of Cr, with a lesser contribution from Al. From the fractional density diagram in Fig. 5b–f, when the Mn atom dissolved into the Fe_22_–Cr_5_–Ni_3_–Al_2_ system, the number of Mn 3d orbital electrons at the Fermi surface energy level increased; however, the number of Mn 3d orbital electrons is lower than that of Fe. Mn occupies the position of the Fe atom, and the charge density around Mn is lower than that of Fe; therefore, the hybridization effect of the Mn, Cr and Ni atoms is weakened; the binding bond between atoms also is weakened, rendering the structure slightly less stable.

### Effect of Mn content on the mechanical properties of low Ni AFA steel

#### Calculation of elastic modulus

According to the parameters in Table 2, the Fe_22_–Cr_5_–Ni_3_–Al_2_ system belongs to the orthogonal crystal system after optimization. A total of nine independent elastic constants are calculated for C11, C12, C13, C22, C23, C33, C44, C55, and C66, as shown in Table 3.

**Table 3:** Elastic constants of the five structural systems.

Elastic constants	C11	C12	C13	C22	C23	C33	C44	C55	C66
Fe_22_–Cr_5_–Ni_3_–Al_2_	47.351	106.929	123.012	53.558	119.218	125.709	156.523	160.754	157.499
Fe_21_–Cr_5_–Ni_3_–Al_2_–Mn	65.450	119.400	116.963	97.254	133.570	123.748	146.471	162.858	154.745
Fe_20_–Cr_5_–Ni_3_–Al_2_–Mn_2_	113.802	121.719	130.655	100.377	112.686	59.580	144.332	156.046	150.898
Fe_19_–Cr_5_–Ni_3_–Al_2_–Mn_3_	134.071	138.222	117.661	88.543	111.152	45.094	148.163	155.269	148.172
Fe_18_–Cr_5_–Ni_3_–Al_2_–Mn_4_	95.812	120.573	127.928	113.109	109.860	108.311	143.168	151.283	142.620

The bulk modulus (B), shear modulus (G), and Young's modulus (Y) of the experimental steels were determined using the Voight–Reuss–Hill (VRH) equation^22^ for all crystalline systems with:

3 $$ B = \frac{1}{9}\left( {C_{11} + C_{22} + C_{33} } \right) + \frac{2}{9}\left( {C_{12} + C_{13} + C_{23} } \right) $$

4 $$ G = \frac{1}{15}\left( {C_{11} + C_{22} + C_{33} - C_{12} - C_{13} - C_{23} } \right) + \frac{1}{5}\left( {C_{44} + C_{55} + C_{66} } \right) $$

5 $$ Y = \frac{9GB}{{G + 3B}} $$

**Figure 6:**
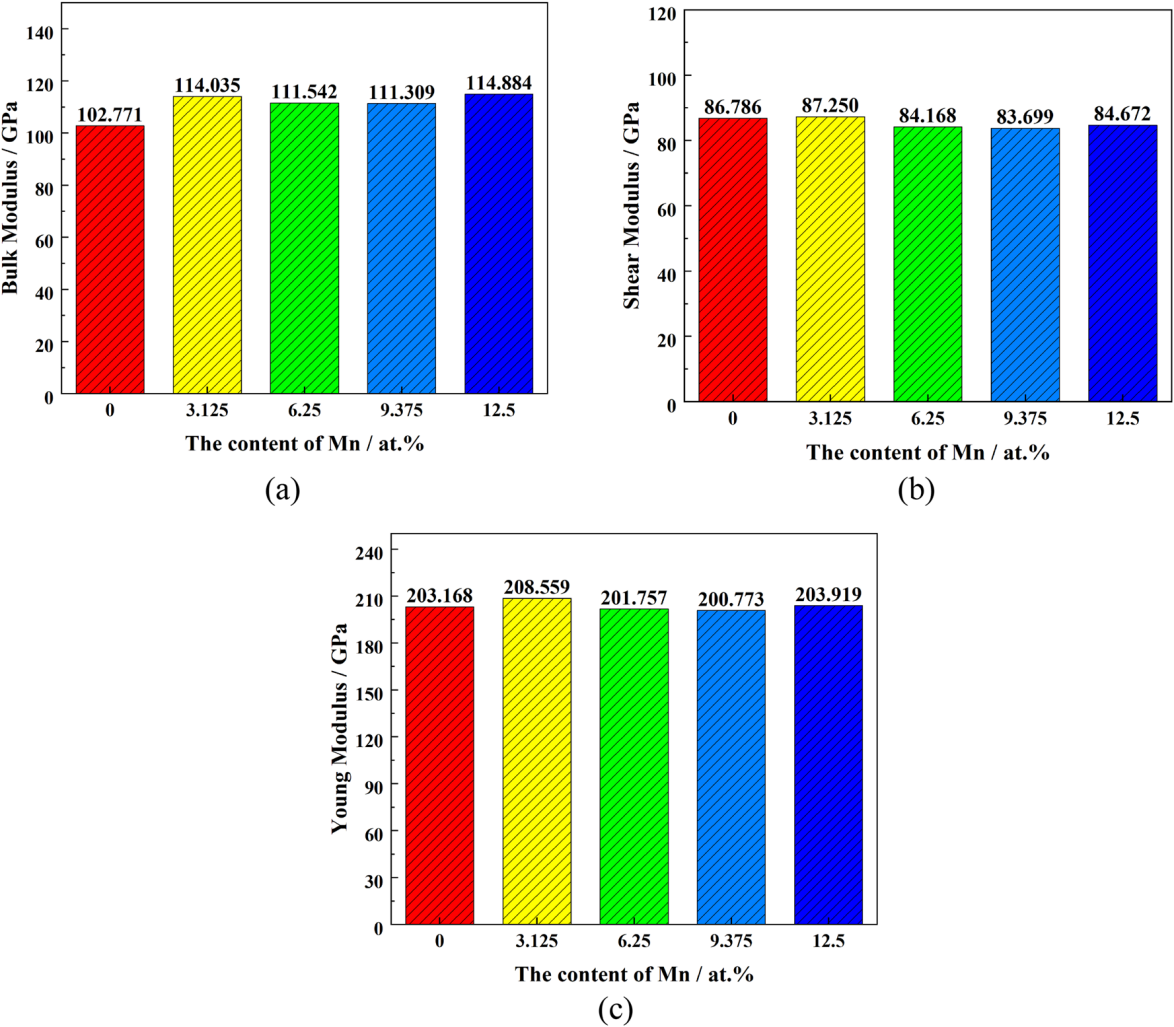
Elastic modulus of the five structural systems: (**a**) bulk modulus (**b**) shear modulus (**c**) Young's modulus.

The values of B, G, and Y for the different systems were calculated according to Eqs. (3) (4) (5) (Fig. 6). With an increase of Mn content in Fe_22_–Cr_5_–Ni_3_–Al_2_, the bulk modulus of the system increased significantly, the shear modulus decreased; Young's modulus was essentially unchanged. The incorporation of Mn in the matrix impacts the steel's ability to resist shear deformation, where stiffness and strength are reduced, but the change is insignificant. Therefore, the solid solution of Mn will reduce the strength of the Fe_22_–Cr_5_–Ni_3_–Al_2_ system, but the effect is slight.

#### Calculation of mechanical properties

**Figure 7:**
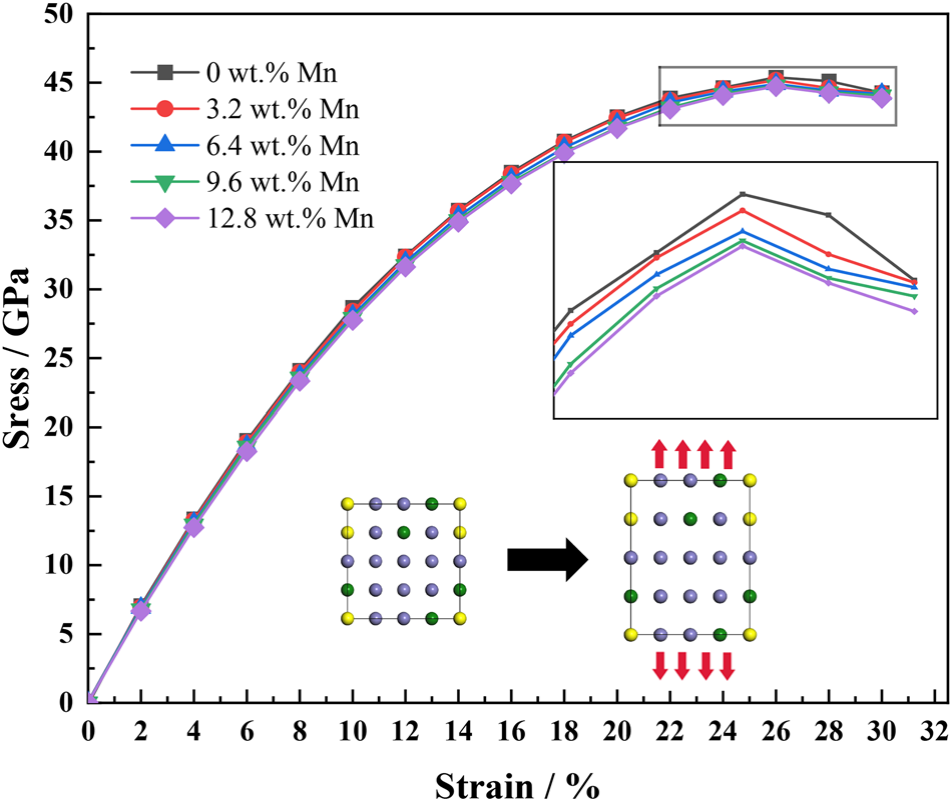
Ideal stress–strain curves of the five structural systems.

The five crystal cells in Fig. 1 were tensioned by applying strain incrementally along the (001) plane of the cell, fixing the z-axis direction of each atom but not fixing the x- and y-axis directions so that the atoms relaxed sufficiently in both the x- and y-directions to obtain the corresponding stresses. The strain was gradually applied until the stresses reached their highest point and then decreased, obtaining the ideal stress–strain curve for each system, as shown in Fig. 7. As the strain increases, the stresses of the five structural cell models increase. When the strain is between 0.26 and 0.28, the stresses reach their maximum values (45.38, 45.18, 44.91, 44.79, and 44.72 GPa, respectively). As the strain increases further, the stresses decrease. With increased Mn content, the highest points of the ideal stress–strain curves for the five systems decrease slightly. The strength of high-Mn AFA steel reduces by about 1% as the Mn content increases from 3.125 at.% (3.2 wt%) to 12.5 at.% (12.8 wt%).

**Figure 8:**
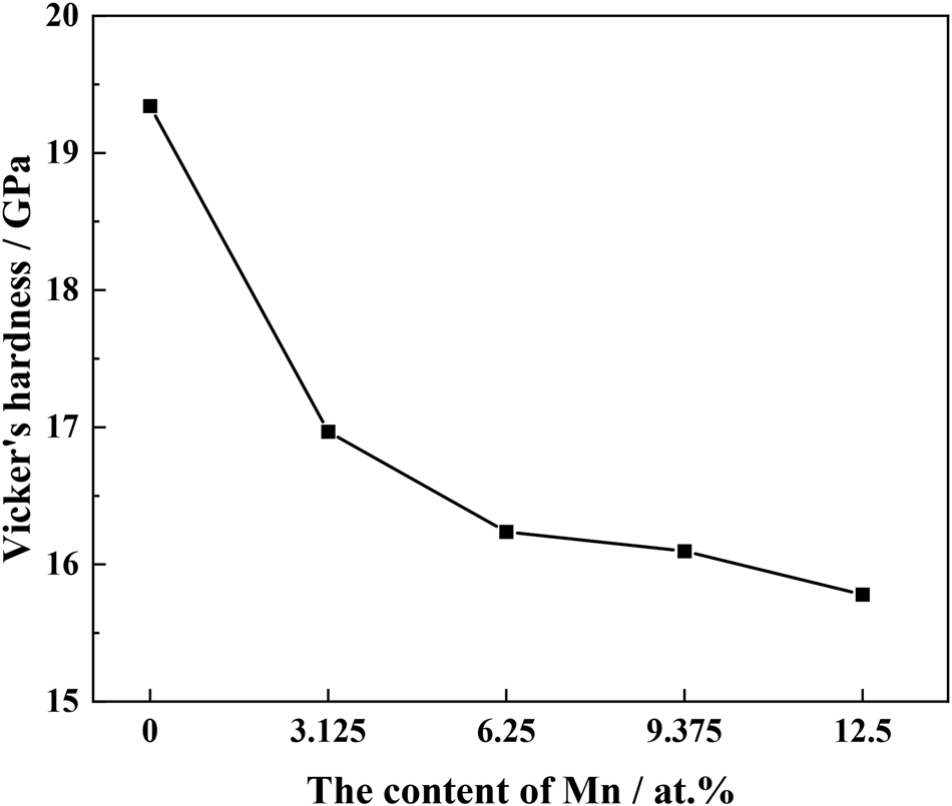
Theoretical microhardness of the five structural systems.

Based on the bulk and shear moduli of the different systems, microhardness was calculated according to the semi-empirical Eq. (6)^23^, and the results are shown in Fig. 8.

6 $$ H_{V} = 2\left( {\frac{G}{{K^{2} }}} \right)^{0.585} - 3 $$

7 $$ K = G/B $$

The microhardness of Fe_22_–Cr_5_–Ni_3_–Al_2_ was the largest, with a value of about 19.4 GPa. When different levels of Mn were incorporated into the system to form Fe_21_–Cr_5_–Ni_3_–Al_2_–Mn, Fe_20_–Cr_5_–Ni_3_–Al_2_–Mn_2_, Fe_19_–Cr_5_–Ni_3_–Al_2_–Mn_3_, and Fe_18_–Cr_5_–Ni_3_–Al_2_–Mn_4_ solid solutions, the microhardness of the newly formed systems all decreased; as the Mn content increased from 0 to 12.8 wt%, the hardness decreased from 19.4 to 15.8 GPa (Fig. 8), which is consistent with Figs. 6, 7. Therefore, the hardness of Al-containing austenite, formed by Mn solid solution in the Fe_22_–Cr_5_–Ni_3_–Al_2_ system, decreases.

**Figure 9:**
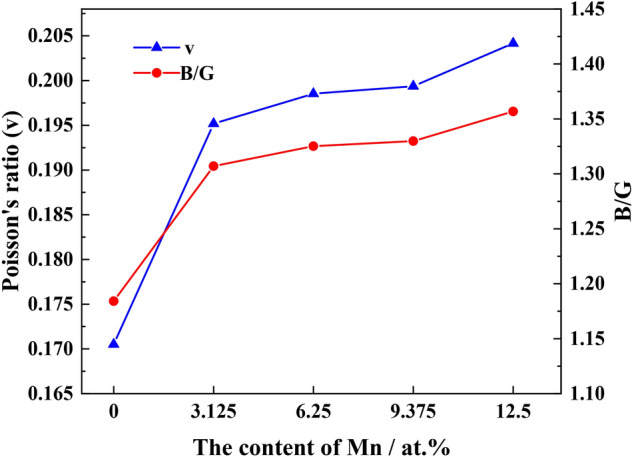
Poisson's ratio (ν) and Pratt's modulus ratio B/G for the five structural systems.

Figure 9 shows Poisson's ratio (ν) and Pratt's modulus ratio (B/G). When the Mn solid solution formed in Fe_22_–Cr_5_–Ni_3_–Al_2_, the Poisson's ratio of the austenite matrix increased with improved elastic properties, i.e., Mn is beneficial in enhancing the elastic properties exhibited by high-Mn AFA steel. An increase in Mn content in the matrix accompanied by the rise in Poisson's ratio from 0.171 to 0.203 with a concomitant increase in Pratt's modulus ratio; B/G for the Fe_18_–Cr_5_–Ni_3_–Al_2_–Mn_4_ system is 1.36, representing the best plasticity. Our findings confirm the beneficial effect of Mn in high-Mn AFA steel in terms of enhancing the elastic properties and plasticity of the austenite matrix.

#### Experimental analysis of mechanical properties

**Figure 10:**
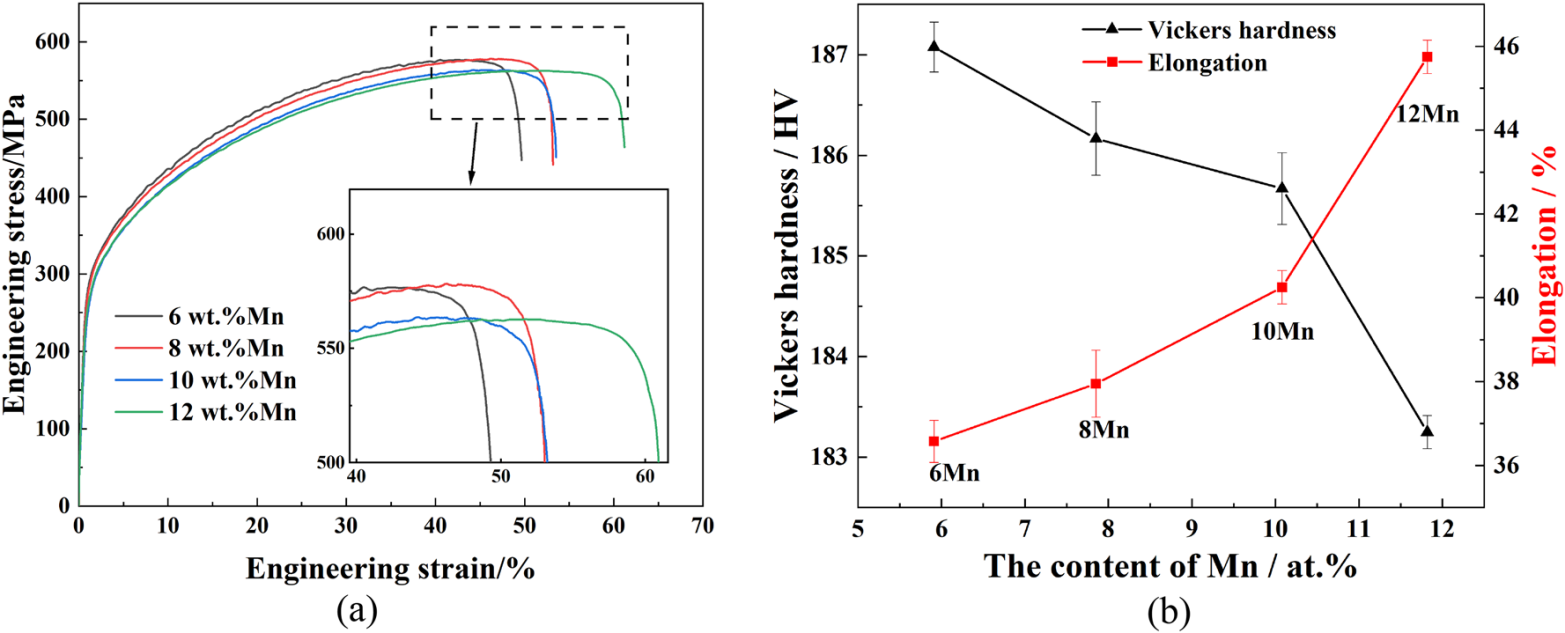
Test results of the mechanical properties of high-Mn AFA steel with different Mn content: (**a**) stress–strain curve (**b**) Vickers hardness and elongation.

Figure 10 shows the mechanical properties of high-Mn AFA steel tested at room temperature with 5.91–11.82 at.% Mn contents (corresponding to 6.03–12.02 wt%, as shown in Table 1). As the Mn content increased, the tensile strength decreased from 581.75 to 565.70 MPa, representing a 2.7% decrease in tensile strength. In addition, the hardness decreased from 187.2 HV to 183.3 HV, and the elongation increased from 36.7 to 45.2%. The increase in Mn content decreased tensile strength and yield strength while increasing elongation and material plasticity but reducing hardness. The results of the elasticity and ideal stress–strain calculations (Figs. 6, 7) also show that at higher Mn content in the Fe_22_–Cr_5_–Ni_3_–Al_2_ system, the strength and hardness of the austenite matrix decrease and plasticity increases. At the Mn content of 8 wt%, the AFA steel has good strength and toughness: a tensile strength of 581.64 MPa, hardness of 186.17 HV, and elongation of 39%. Thus, after adding 8.0 wt% Mn to the high-Mn AFA steel with 10 wt% Ni content, the austenite is stable, and the mechanical properties are optimal.

## Conclusion

1. Adding Mn to low Ni-AFA steel increases the unit cell volume with an accompanying increase in the absolute value of formation energy; the austenite formed more easily. The austenitic matrix binding energy decreases and remains negative, indicating austenite stability. As the Mn content increases from 3.2 to 12.8 wt%, the system's bulk modulus (B) rises significantly, and the shear modulus (G) falls. In addition, the system's strength and hardness decrease, and the Poisson ratio of the austenite matrix increases with improved elasticity; the system has excellent plasticity with an increase in the B/G.
2. Addition of Mn does not change the energy level structure of the Fe_22_–Cr_5_–Ni_3_–Al_2_ system. All the systems have a non-zero number of electrons at the Fermi energy level and exhibit metallic properties. At different Mn solid solution content in Fe_22_–Cr_5_–Ni_3_–Al_2_, the number of electrons at the Fermi energy level increases first and then decreases. An appropriate amount of Mn atoms can improve the electrochemical stability of the matrix. As the content of Mn increases to 9.6 wt%, the electrochemical stability of the matrix gradually increases. However, with Mn further increasing to 12.8 wt%, the stability of the matrix decreases.
3. When the Mn content increases from 6.03 to 12.02 wt%, the tensile strength and microhardness decrease from 581.75 to 565.70 MPa and 187.2 to 183.3 HV, respectively. Accordingly, the elongation increases from 36.7 to 45.2%. At a Mn content of 8 wt%, the overall mechanical properties of the high-Mn AFA steel are optimal, with a tensile strength of 581.64 MPa, a hardness of 186.17 HV, and an elongation of 39%.

## Data Availability

The datasets generated during and analyzed during the current study are available from the corresponding author on reasonable request.
